# Clinical Use of Cinacalcet in MEN1 Hyperparathyroidism

**DOI:** 10.1155/2010/906163

**Published:** 2010-05-26

**Authors:** V. J. Moyes, J. P. Monson, S. L. Chew, S. A. Akker

**Affiliations:** Department of Endocrinology, St. Bartholomew's Hospital, London EC1A 7BE, UK

## Abstract

*Background*. Management of multiple-endocrine neoplasia type 1- (MEN1-) associated hyperparathyroidism is associated with high recurrence rates and high surgical morbidity due to multiple neck explorations. Cinacalcet, a calcimimetic agent licensed for the treatment of secondary hyperparathyroidism and parathyroid carcinoma, may provide a medical alternative for the management of these complex patients. *Methods*. A prospective audit was performed of eight patients; three males and five females, aged 20–38 at diagnosis. Two patients commenced cinacalcet as primary treatment and six had previous surgery. Six patients had complications of hyperparathyroidism: renal calculi, renal dysfunction, and reduced bone mineral density. All were commenced on cinacalcet 30 mg bd for MEN1 associated hyperparathyroidism; doses were subsequently reduced to 30 mg od in four patients. *Results*. Significant reductions were observed in serum calcium and PTH measurements. Serum calcium reduced by a median of 0.35 mmol/L (*P* = .012 Wilcoxon Signed Rank). Serum PTH levels decreased by a median of 5.05 pmol/L (*P* = .012). There was no change in urine calcium. Duration ranged from 10–35 months with maintenance of control. Cinacalcet was well tolerated by six patients; one experienced nausea and one experienced diarrhoea. *Conclusion*. Cinacalcet is an effective and well-tolerated medical treatment for the management of complex primary hyperparathyroidism.

## 1. Introduction

Primary hyperparathyroidism (PHPT) in Multiple Endocrine Neoplasia Type I (MEN1) has a penetrance rate of nearly 100% by the age of 50 years [1]. The underlying pathology in MEN1 PHPT differs from sporadic disease; parathyroid hyperplasia is present rather than a monoclonal adenoma. Differences in the natural history are evident; patients present at a younger age (20–25 years) with early onset of complications for example, affected women have a reduced bone mineral density (BMD) by the age of 35 years [2]. Effective management of MEN1 PHPT is advisable to reduce complication rates; a significant reduction in BMD has been observed in MEN1 patients with uncontrolled PHPT compared to those with controlled PHPT [2]. Surgical cure of MEN1 PHPT is difficult to achieve; multiple gland hyperplasia, involvement of more than 4 glands and ectopic locations such as the mediastinum dictate the need for thorough preoperative localisation. There remains controversy over the optimal surgical intervention; total parathyroidectomy necessitates the use of long-term activated vitamin D and the potential complication of hypercalciuria. Whilst subtotal parathyroidectomy avoids this, further hyperplasia is likely to occur in residual parathyroid tissue; recurrence rates of 50% were reported at 8–12 years post successful subtotal parathyroidectomy [3]. Multiple operations are often required with a consequent increase in surgical morbidity. 

Cinacalcet is a potential medical alternative; by allosterically modulating the calcium sensing receptor on the PTH cell, it increases sensitivity to extracellular calcium and down-regulates PTH secretion. It is licensed for the management of secondary hyperparathyroidism and parathyroid carcinoma [4, 5]. In primary hyperparathyroidism, reductions in serum calcium and to a lesser extent serum PTH levels have been demonstrated [6, 7], but the likelihood of surgical cure in sporadic disease usually avoids the need for additional medical intervention.

## 2. Patients and Methods

We reviewed the clinical data of nine patients treated with cinacalcet for MEN-1 PHPT with institutional board approval. The cohort consisted of three males, five females, aged 20–38 years at diagnosis (Table 1). All had clinical MEN1 with positive menin mutation. All patients had hypercalcaemia and elevated serum PTH levels confirmed at diagnosis. Six had previous noncurative surgical intervention; two had multiple prior neck explorations. Two patients were unable to undergo surgery; one due to severe learning difficulties and the second due to an anxiety disorder, and cinacalcet was commenced as primary treatment. 

Pretreatment serum calcium levels ranged 2.62–2.91 mmol/L, median 2.76 mmol/L (reference range 2.15–2.65 mmol/L), and PTH levels ranged 4.8–36.5 pmol/L, median 15.9 pmol/L (reference range 1.3–6.8 pmol/L). All patients deficient in 25-hydroxy vitamin D were commenced on supplements and achieved levels within the laboratory reference range prior to commencement of cinacalcet. Six out of eight patients exhibited complications of PHPT: reduced BMD in five patients, (T score of less than −2.5), renal calculi in four patients, and renal dysfunction in four patients (>25% reduction in creatinine clearance). Two patients had no complications of hyperparathyroidism but were under the age of 50; all patients therefore fulfilled criteria for treatment as determined by the Consensus on the management of asymptomatic primary hyperparathyroidism 2002 [8]. 

Cinacalcet was generously provided by Amgen on a compassionate-use basis to the hospital for no charge. After a product license was obtained in the UK for use in renal failure-related hyperparathyroidism, it was prescribed on an off label basis at 30 mg twice daily. The dose was subsequently reduced in four patients: two due to clinical response and two due to side effects. Due to the pharmacokinetics of cinacalcet, all serum calcium and PTH measurements were taken predose according to a defined protocol. As steady state drug levels are achieved after 7 days, results of the first clinical assessment (three months postcommencement) were used for comparison.

## 3. Statistical Analysis

Statistical significance was accepted at a *P* value <.05. All variables studied were normally distributed. The nonparametric Wilcoxon Signed Ranks Test was performed for the comparison of the variables between the studied groups because of the small sample size.

Analysis was performed using SPSS (version 11.01; Spss, Inc., Chicago, IL) for W indows XP (Microsoft Corp).

## 4. Results

**Figure 1:**
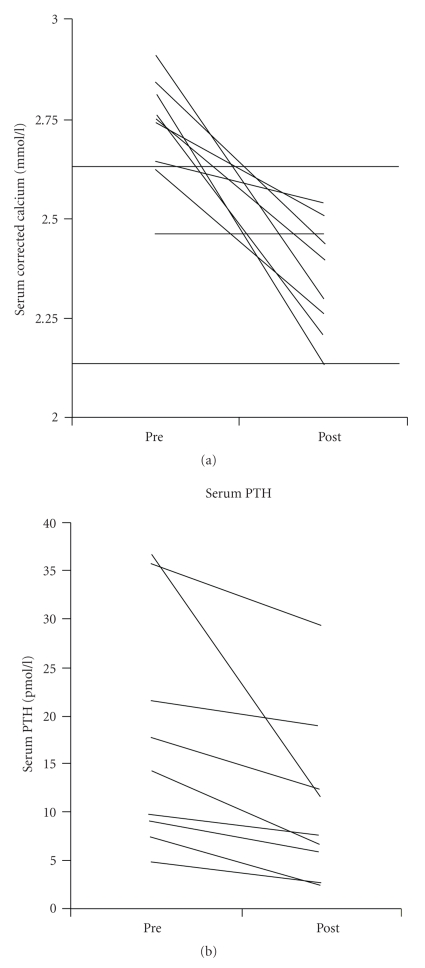
Change in biochemical parameters pre- and post commencement of cinacalcet. These graphs demonstrate the change in serum calcium and serum PTH pre- and post commencement of cinacalcet. Each line represents an individual patients' data. The grey dotted line indicates the normal reference range.

All patients achieved normocalcaemia on cinacalcet with a median posttreatment serum corrected calcium of 2.35 mmol/L, range of 2.13–2.54 mmol/L. Serum calcium reduced by a median of 0.68 mmol/L, range of 0.10–0.68 mmol/L, and was statistically significant (*P* = .012) (Figure 1(a)). Serum PTH reduced by median of 5.05 pmol/L, range of 0.7–24.8 pmol/L, (*P* = .012) (Figure 1(b)). Analyses were repeated following removal of a potential outlier (patient number 8) to confirm statistical significance; this confirmed a significant reduction in serum PTH (*P* = .018). A significant increase was noted in serum phosphate (*P* = .012) but no change was found in 24-hour urinary calcium measurements (Table 2).

A comparison was made between pre- and 2 hours post cinacalcet PTH measurements for the 5 individuals in whom results were available; no significant difference was detected (*P* = .345).

 Cinacalcet was well tolerated by six patients; two experienced gastro-intestinal side effects and one subsequently discontinued cinacalcet. Duration of treatment was 10–35 months with maintenance of biochemical control achieved in seven patients; the eighth patient defaulted from clinical follow up. Incomplete clinical data precluded the statistical comparison of disease end points; however, no difference was noted in BMD for the five patients reassessed. Patients with a history of renal calculi had been successfully treated previously; none demonstrated recurrent stone formation.

## 5. Discussion

Our data demonstrate that cinacalcet is effective at significantly lowering serum calcium and PTH levels but insufficient data were available to draw conclusions regarding disease end points. Control of serum calcium and PTH in MEN1 PHPT patients treated with surgery is associated with an increase in BMD [2] which suggests that aiming for biochemical control is an appropriate therapeutic strategy in these patients. Where surgery is not possible or has failed to control MEN1 PHPT, cinacalcet does provide an alternative therapeutic option. Existing medical options are limited; bisphosphonates are effective in improving bone density but have no significant effect on serum corrected calcium or PTH levels [9]. Faggiano et al. [10] recently demonstrated the effectiveness of long acting octreotide in the management of MEN1 patients with primary hyperparathyroidism and duodeno-pancreatic neuroendocrine tumour. However, long-term adverse metabolic sequelae are likely to outweigh its benefits for primary hyperparathyroidism alone [11–13].

We have demonstrated a more impressive reduction in serum PTH (median 35.5%, range 3.25–67.9%) compared to studies of cinacalcet use in sporadic PHPT (mean reduction 7.6%) [6]. Timing of PTH samples in relation to cinacalcet dosing does account for potential variability in results; a reduction of 37% has been demonstrated at 2 hours postdose, with recovery of predose levels by 8 hours [6]. Such pharmacodynamics were not evident in our cohort; no significant difference was detected between predose and 2-hour postdose PTH measurements for the five patients in whom results were available. All samples within our cohort were taken predose as per standard clinical practice. The reasons for the enhanced PTH response seen in our cohort are not clear; we postulate that the underlying pathology may be of importance. MEN1 hyperparathyroidism is invariably due to chief cell hyperplasia rather than a monoclonal adenoma. It may be possible that the expression of calcium sensing receptors differs between the two phenotypes and this may influence biochemical response to cinacalcet, although data to support this are currently lacking.

Previously published data highlighted the theoretical concern of increased urinary calcium excretion with cinacalcet use, due to the direct action at the calcium sensing receptors in the kidney. The overall reduction in serum calcium appears to counteract this effect and significant changes in urinary calcium excretion have not been demonstrated in primary hyperparathyroidism [6]. In view of the theoretical concern, it is the authors' policy to measure 24-hour urine calcium levels as part of the patient's annual clinical assessment in addition to regular assessment of serum calcium, phosphate, and PTH measurements. 

Effective control of serum calcium and PTH levels in MEN1 PHPT is an appropriate therapeutic strategy to reduce the early onset of associated complications. Where surgery has been noncurative or is not possible, cinacalcet may provide a medical alternative; this is supported by our study and a recent case report of a 30-year-old woman who maintained biochemical control after 1 year of cinacalcet for management of MEN1 hyperparathyroidism [14]. Longer-term prospective studies are needed to further evaluate clinical effectiveness, safety, and tolerability.

## Figures and Tables

**Table 1:** Summary of clinical details, demographics and biochemical data for the cohort studied. This table summarises the details regarding the diagnosis of MEN1, parathyroid disease, and biochemical data pre- and post commencement of cinacalcet. A significant reduction in serum corrected calcium and serum PTH was determined. Bracketed () results indicate PTH levels taken 4 hours post dose.

Pt	Age/ M/F	Diagnoses	Prev PTH surgery (No)	Number Glands removed	Complications			Serum corr-Calcium mmol/L		Serum PTH pmol/L	
					Low BMD	Renal Calculi	Low GFR	Pre	Post	Pre	Post
1	35 F	PTH, PRL, NF Islet cell	0	n/a	N	N	N	2.62	2.26	35.8	29.4 ** (41.7)**

2	40 M	PTH, PRL	1	3	N	Y	N	2.64	2.54	17.6	12.4

3	58 M	PTH, Gastrinoma	2	4	Y	N	Y	2.81	2.13	14.1	6.8 ** (12.8)**

4	52 M	PTH, NF Islet cell	1	3	Y	N	N	2.76	2.21	4.8	2.8 ** (3.1)**

5	53 F	PTH, NFPA, Gastrinoma	4	2	Y	Y	Y	2.74	2.51	9.8	7.7 ** (3.8)**

6	26 F	PTH, NF Islet cell	1	3	N	N	N	2.84	2.44	7.3	2.4 **(2.4)**

7	46 F	PTH, Gastrinoma	0	n/a	Y	Y	Y	2.91	2.30	36.5	11.7

8	38 F	PTH, PRL, Gastrinoma	1	2	Y	Y	N	2.75	2.40	21.5	18.6

Key: M: Male; F: female; PTH: Hyperparathyroidism; PRL: prolactinoma; NF Islet Cell: Non functioning Islet Cell tumour; BMD: Bone Mineral Density; n/a not applicable; No: number; GFR:Glomerular Filtration Rate.

**Table 2:** Biochemical results pre- and post commencement of cinacalcet with nonparametric statistical analyses.

	Premedian (Range)	Postmedian (Range)	*P*-value
Serum Calcium (mmol/L)	2.76 (2.62–2.91)	2.35 (2.13–2.54)	.012

Serum PTH (pmol/L)	15.85 (4.8–36.5)	9.7 (2.4–29.4)	.012

Serum Phosphate (mmol/L)	0.91 (0.77–0.99)	1.04 (0.78–1.31)	.012

Urine Calcium (mmol/24 hours)	5.02 (2.54–7.79)	3.35 (1.98–5.6)	.144

## References

[1] Brandi ML, Gagel RF, Angeli A (2001). Consensus: guidelines for diagnosis and therapy of MEN type 1 and type 2. *Journal of Clinical Endocrinology and Metabolism*.

[2] Burgess JR, David R, Greenaway TM, Parameswaran V, Shepherd JJ (1999). Osteoporosis in multiple endocrine neoplasia type 1. Severity, clinical significance, relationship to primary hyperparathyroidism, and response to parathyroidectomy. *Archives of Surgery*.

[3] Marx S, Scriver CR, Beaudet AL, Sly WS, Valle D (2001). Multiple endocrine neoplasia type 1. *The Metabolic and Molecular Bases of Inherited Disease*.

[4] Moe SM, Cunningham J, Bommer J (2005). Long-term treatment of secondary hyperparathyroidism with the calcimimetic cinacalcet HCl. *Nephrology Dialysis Transplantation*.

[5] Silverberg SJ, Rubin MR, Faiman C (2007). Cinacalcet hydrochloride reduces the serum calcium concentration in inoperable parathyroid carcinoma. *Journal of Clinical Endocrinology and Metabolism*.

[6] Peacock M, Bilezikian JP, Klassen PS, Guo MD, Turner SA, Shoback D (2005). Cinacalcet hydrochloride maintains long-term normocalcemia in patients with primary hyperparathyroidism. *Journal of Clinical Endocrinology and Metabolism*.

[7] Shoback DM, Bilezikian JP, Turner SA, McCary LC, Guo MD, Peacock M (2003). The calcimimetic cinacalcet normalizes serum calcium in subjects with primary hyperparathyroidism. *Journal of Clinical Endocrinology and Metabolism*.

[8] Bilezikian JP, Potts JT, Fuleihan GE-H (2002). Summary statement from a workshop on asymptomatic primary hyperparathyroidism: a perspective for the 21st century. *Journal of Clinical Endocrinology and Metabolism*.

[9] Rossini M, Gatti D, Isaia G, Sartori L, Braga V, Adami S (2001). Effects of oral alendronate in elderly patients with osteoporosis and mild primary hyperparathyroidism. *Journal of Bone and Mineral Research*.

[10] Faggiano A, Tavares LB, Tauchmanova L (2008). Effect of treatment with depot somatostatin analogue octreotide on primary hyperparathyroidism (PHP) in multiple endocrine neoplasia type 1 (MEN1) patients. *Clinical Endocrinology*.

[11] Lancranjan I, Atkinson AB (1999). Results of a European multicentre study with Sandostatin LAR in acromegalic patients. *Pituitary*.

[12] Lembcke B, Creutzfeldt W, Schleser S, Ebert R, Shaw C, Koop I (1987). Effect of the somatostatin analogue Sandostatin (SMS 201-995) on gastrointestinal, pancreatic and biliary function and hormone release in normal men. *Digestion*.

[13] Moschetta A, Stolk MFJ, Rehfeld JF (2001). Severe impairment of postprandial cholecystokinin release and gall-bladder emptying and high risk of gallstone formation in acromegalic patients during Sandostatin LAR. *Alimentary Pharmacology and Therapeutics*.

[14] Falchetti A, Cilotti A, Vagelli L (2008). A patient with MEN1-associated hyperparathyroidism, responsive to cinacalcet. *Nature Clinical Practice Endocrinology and Metabolism*.

